# Metabolic and Candidate Genetic Factors Associated with Overweight/Obesity in Patients of Kazakh Ethnicity with Psoriasis: A Cross-Sectional Study

**DOI:** 10.3390/medicina62081448

**Published:** 2026-07-26

**Authors:** Botagoz Zhumabekova, Gulnara Svyatova, Gulsum Askarova, Zhanay Akanov, Mir-Ali Baltabaev, Anastassiya Ge, Auyeskhan Dzhumabekov, Gulbanu Berdiyarova, Ayash Baisultanova, Gaukhar Issakova

**Affiliations:** 1 Department of Epidemiology, Evidence-Based Medicine and Biostatistics, Kazakhstan’s Medical University “KSPH”, Almaty 050060, Kazakhstan; dr.jumabekov@gmail.com; 2 Scientific Department of the Center for Molecular Medicine, Center for Molecular Medicine, Almaty 050026, Kazakhstan; gsvyatova1@mail.ru; 3 Department of Dermatovenereology, Kazakhstan’s Medical University “KSPH”, Almaty 050060, Kazakhstan; gkaskarova1@gmail.com; 4 Public Foundation “Kazakhstan Society for the Study of Diabetes”, Almaty 050026, Kazakhstan; aaaendoclinic@gmail.com; 5 Department of Dermatovenereology and Phthisiology, Kyrgyz-Russian Slavic University, Bishkek 720000, Kyrgyzstan; mirali.baltabaev@gmail.com; 6 Center for Molecular Medicine LLP, Almaty 050026, Kazakhstan; geanastasiya@gmail.com; 7 Department of Anesthesiology, Critical Care and Emergency Medicine, Kazakhstan’s Medical University “KSPH”, Almaty 050060, Kazakhstan; berdiyarovagulbanu@gmail.com; 8 Department of Therapy with a Course in Gerontology, Kazakhstan’s Medical University “KSPH”, Almaty 050060, Kazakhstan; baisultanova.ayash@gmail.com; 9 Department of Gastroenterology, Kazakhstan’s Medical University “KSPH”, Almaty 050060, Kazakhstan; gauharissakova@gmail.com

**Keywords:** psoriasis, overweight, obesity, inflammatory biomarkers, rs1558902, rs696574, rs10968110, genetic polymorphisms, *FTO*, ZTV, CALCRL

## Abstract

*Background and Objectives*: Psoriasis is a chronic immune-mediated inflammatory disease frequently accompanied by obesity and cardiometabolic disturbances. This study evaluated metabolic, inflammatory, and genetic correlates of overweight/obesity in ethnic Kazakh patients with psoriasis and explored potential multilocus genetic interactions. *Materials and Methods*: This study included 150 patients with clinically confirmed psoriasis, comprising 75 patients with overweight/obesity and 75 non-overweight/obese individuals frequency-matched by age and sex; because group sizes were fixed by design rather than sampled in proportion to prevalence, the reported odds ratios reflect within-sample associations rather than population-level risk estimates. Clinical, anthropometric, biochemical, inflammatory, and genetic data were collected using standardized protocols. Multivariable logistic regression was used to assess clinical, metabolic, and inflammatory correlates of overweight/obesity. Associations between three candidate polymorphisms, rs1558902 in *FTO*, rs696574 in *CALCRL*, and rs10968110 in *ZTV*, were evaluated. *Results:* In the multivariable model adjusted for age and sex, higher triglyceride levels (adjusted OR 4.73, 95% CI 1.36–18.57), higher HbA1c (adjusted OR 1.71, 95% CI 1.09–2.91), and lower HDL-C levels (adjusted OR 0.36, 95% CI 0.18–0.66) were independently associated with overweight/obesity. In genetic analyses, rs10968110 in *ZTV* showed significant associations with overweight/obesity in genotype, additive trend, allelic, and dominant models, while the recessive model was not significant. The *FTO* rs1558902 variant was also significantly associated with overweight/obesity, with the strongest support observed under additive, allelic, and dominant assumptions. In contrast, rs696574 in *CALCRL* was not significantly associated with overweight/obesity. Exploratory MDR analysis identified rs10968110 as the most stable single-locus model, whereas higher-order models showed only limited additional discriminatory value and lower cross-validation consistency. *Conclusions:* Among ethnic Kazakh patients with psoriasis, overweight/obesity was associated with an adverse cardiometabolic profile characterized by elevated triglycerides, higher HbA1c, and reduced HDL-C. Candidate genetic findings suggest that *ZTV* rs10968110 and *FTO* rs1558902 may be associated with overweight/obesity susceptibility, although these results should be considered exploratory pending validation in larger independent cohorts. These findings support integrated cardiometabolic assessment in psoriasis care and further investigation of genetic susceptibility to obesity in this population.

## 1. Introduction

Psoriasis is a chronic immune-mediated inflammatory disease that is increasingly recognized as a systemic condition with significant metabolic comorbidities. Its prevalence varies by region, with the highest rates reported in Australasia (1.99%) and Western Europe (1.92%), and by age, with a higher prevalence observed among adults [1]. The disease demonstrates a bimodal distribution of onset, with peaks at approximately 15–20 and 55–60 years of age [2]. Beyond its dermatologic manifestations, psoriasis has been consistently associated with obesity, insulin resistance, dyslipidemia, and an increased risk of cardiovascular disease [3]. The coexistence of psoriasis and metabolic disorders suggests shared pathophysiological mechanisms, including chronic low-grade inflammation, immune dysregulation, and alterations in lipid metabolism.

Obesity is highly prevalent among patients with psoriasis and may influence both disease onset and progression [4,5]. Excess adiposity has been associated with increased production of pro-inflammatory cytokines, such as tumor necrosis factor alpha (TNF-α) and interleukin-6 (IL-6), which are also central to the pathogenesis of psoriasis [6,7,8,9]. Higher body mass index (BMI) has been linked to greater psoriasis severity in some studies [10]; however, the evidence remains inconsistent. The relative contributions of metabolic versus inflammatory pathways to overweight/obesity in this population are not fully understood. Furthermore, although metabolic abnormalities are well documented in psoriasis, the specific biochemical drivers of overweight/obesity—particularly across diverse populations—require further clarification.

In addition to metabolic and inflammatory factors, genetic predisposition may play a role in the development of obesity among patients with psoriasis [11,12]. Variants in genes involved in energy balance, vascular signaling, and metabolic regulation, such as *FTO*, *CALCRL*, and *ZTV*, have been implicated in obesity-related traits in general populations [11,13]. However, their relevance in psoriasis remains poorly characterized. Moreover, the extent to which gene–gene interactions contribute to overweight/obesity risk, beyond individual variant effects, is not well established, particularly in Central Asian populations.

Therefore, the present study aimed to evaluate metabolic, inflammatory, and genetic determinants of overweight/obesity in patients with psoriasis. Specifically, we investigated whether dyslipidemia and glycemic markers are independently associated with overweight/obesity, assessed the role of selected candidate polymorphisms, and explored potential gene–gene interactions using multifactor dimensionality reduction analysis. By integrating clinical, biochemical, and genetic data, this study seeks to provide a more comprehensive understanding of the factors contributing to overweight/obesity in psoriasis and to inform future risk stratification and management strategies.

## 2. Materials and Methods

### 2.1. Study Design and Study Population

This study included 150 ethnic Kazakh patients with clinically confirmed psoriasis who were recruited between June 2025 and January 2026 from three institutions in Almaty, Kazakhstan. Clinical assessments and routine laboratory testing were conducted at the Almaty City Dermatovenereologic Dispensary, cytokine analyses at ILAB 100+, and genotyping of obesity-related polymorphisms at the Center for Molecular Medicine LLP. Although clinical, biochemical, and genetic data were collected using a cross-sectional protocol (all measurements obtained at a single time point, without follow-up), participants were selected using a fixed 1:1 ratio of overweight/obese to non-overweight/obese individuals, frequency-matched by age and sex. This sampling approach resembles a frequency-matched case–control design with respect to the overweight/obesity outcome. Group sizes therefore do not reflect the prevalence of overweight/obesity among psoriasis patients at the participating institutions, and the odds ratios reported in this study should be interpreted as measures of association within this matched sample rather than as estimates of population-level risk or prevalence (see Section 4, Limitations).

Patients with overweight/obesity were frequency-balanced by age and sex with psoriasis patients without overweight/obesity from the same ethnic background. Clinical, anthropometric, biochemical, immunological, and genetic data were collected using standardized assessment protocols.

Participants were classified according to body mass index (BMI) into two groups: those with overweight/obesity and those without. Overweight/obesity was defined as BMI ≥ 28 kg/m^2^, based on cutoff values commonly applied in Asian populations [14,15]. This threshold is lower than the conventional World Health Organization (WHO) reference for European populations and reflects differences in body composition and cardiometabolic risk profiles observed among Asian populations [16]. Given that ethnic Kazakhs are a Central Asian population, which shares anthropometric and metabolic characteristics with Asian groups, the use of this lower BMI threshold was considered appropriate for this study.

### 2.2. Study Outcomes

The primary outcome was overweight/obesity, defined as a binary variable based on BMI. Participants with a BMI < 28 kg/m^2^ were classified as the reference group (non-overweight/non-obese), whereas those with a BMI≥ 28 kg/m^2^ were classified as having overweight/obesity.

Independent variables were selected a priori based on clinical relevance and existing evidence linking psoriasis with metabolic and inflammatory dysregulation. These included sociodemographic characteristics (age, modeled as a continuous variable in years, and gender [male/female]), anthropometric and clinical measures (waist circumference in centimeters and psoriasis severity assessed using the Psoriasis Area and Severity Index [PASI]), biochemical markers (fasting glucose, glycated hemoglobin [HbA1c], total cholesterol, triglycerides, low-density lipoprotein cholesterol [LDL-C], and high-density lipoprotein cholesterol [HDL-C]), and inflammatory markers (tumor necrosis factor alpha [TNF-α], interleukin-6 [IL-6], and interleukin-10 [IL-10]). Due to right-skewed distributions, triglycerides and inflammatory markers (TNF-α, IL-6, and IL-10) were log-transformed prior to inclusion in regression models. Information on systemic psoriasis-directed treatment, disease duration, smoking status, alcohol consumption, physical activity, and dietary habits was not systematically collected as part of the study protocol and therefore could not be included as covariates in the regression models. This is addressed as a study limitation in Section 4.

### 2.3. Genotyping and SNP Characterization

Three candidate single nucleotide polymorphisms (SNPs)—rs1558902 in the *FTO* gene, rs696574 in the *CALCRL* gene, and rs10968110 in the *ZTV* gene—were selected for replication genotyping in the Kazakhstani population. These variants were chosen because they had previously been reported in genome-wide association studies or candidate-gene studies as being associated with obesity, adiposity-related traits, or metabolic phenotypes in other studied populations. Given the close biological links between obesity, systemic inflammation, cardiometabolic dysregulation, and psoriasis severity, these SNPs were considered biologically plausible candidates for evaluating genetic susceptibility patterns in the present study population. Selection also considered allele-frequency distributions to ensure sufficient variability for genetic analysis within the study sample.

Peripheral venous blood samples were collected into EDTA-containing tubes under standardized conditions. Genomic DNA was isolated using an automated magnetic bead-based extraction method on the Prepito^®^ platform (PerkinElmer, Turku, Finland) with a commercially available DNA purification kit, following the manufacturer’s protocol. DNA concentration and purity were assessed using a NanoDrop spectrophotometer (Thermo Fisher Scientific, Waltham, MA, USA) by measuring absorbance at 260 and 280 nm. The A260/A280 ratio was used to evaluate DNA purity, with values approximating 1.8 considered indicative of high-quality DNA suitable for downstream genotyping. Extracted DNA was stored at −40 °C under nuclease-free conditions until analysis.

Genotyping was performed using real-time polymerase chain reaction (PCR) with allele-specific TaqMan^®^ assays on the StepOnePlus™ system (Applied Biosystems, Foster City, CA, USA). All reactions were conducted in accordance with standardized protocols, including appropriate controls and reagent conditions. Genotype calls were determined based on fluorescence signal clustering, and samples with ambiguous or inconsistent clustering were reanalyzed to ensure accuracy.

Quality control procedures included assessment of genotyping call rates, replicate testing, and evaluation of Hardy–Weinberg equilibrium (HWE). A subset of samples (5%) was randomly selected for repeat genotyping, yielding complete concordance. SNPs with call rates below 95% or significant deviation from HWE were excluded from further analysis. Genotype distributions and allele frequencies were calculated for each SNP (Supplemental Table S1).

For statistical analysis, each SNP was evaluated under additive, dominant, and recessive genetic models. The additive model was coded according to the number of effect alleles (0, 1, or 2), the dominant model compared carriers of at least one effect allele with non-carriers, and the recessive model compared homozygous carriers of the effect allele with all others. The designation of effect alleles was guided by prior literature and biological relevance.

### 2.4. Statistical Analysis

Continuous variables were summarized as means and standard deviations (SD), while categorical variables were presented as frequencies and percentages. Between-group comparisons (overweight/obesity vs. non-overweight) were conducted using the Mann–Whitney U test for continuous variables and chi-square or Fisher’s exact tests for categorical variables, as appropriate. Univariable logistic regression models were used to evaluate the association between each independent variable and overweight/obesity. The multivariable model included clinically relevant variables selected a priori. Candidate variables for the multivariable model were selected a priori based on established clinical and biological relevance to psoriasis-associated metabolic disturbance, rather than through stepwise or other data-driven selection procedures. Prior to model fitting, pairwise correlations among candidate predictors were examined, and multicollinearity was assessed using variance inflation factors (VIFs). Adjusted odds ratios (aORs) with 95% confidence intervals (CIs) were reported.

Associations between candidate SNPs and overweight/obesity were evaluated using Pearson’s chi-square test under genotype (GENO), allelic, dominant (DOM), and recessive (REC) models, while the Cochran–Armitage trend test was applied for the trend model. Gene–gene interactions were explored using multifactor dimensionality reduction (MDR) with a 10-fold cross-validation framework. All possible one-, two-, and three-locus combinations of the candidate SNPs were evaluated. The best models were selected based on the highest balanced accuracy and cross-validation consistency and were subsequently validated in the full dataset.

Analyses were performed using R statistical software (version 4.5.1, dated 13 June 2025, R Foundation for Statistical Computing, Vienna, Austria) and PLINK version 1.9 (Center for Human Genetic Research, Massachusetts General Hospital, Boston, MA, USA) [17]. All statistical tests were two-sided, and statistical significance was defined as *p* < 0.05 unless otherwise specified. Data analyzed in the present study are presented as Supplementary Table S2.

### 2.5. Ethics Statement

This study was conducted in accordance with the principles of the Declaration of Helsinki. Ethical approval was obtained from the Local Ethics Committee of Kazakhstan Medical University “Higher School of Public Health” (approval number: 8; date: 6 May 2025). Written informed consent was obtained from all study participants.

## 3. Results

Baseline characteristics stratified by BMI category are presented in Table 1. A total of 150 patients with psoriasis were included in the analysis, of whom 75 (50.0%) were participants with overweight/obesity. Consistent with the frequency-balanced study design, age and sex distributions did not differ significantly between the BMI groups (*p* = 0.3 and *p* = 0.2, respectively). As expected, anthropometric measures differed markedly: patients with overweight/obesity had substantially higher weight, BMI, and waist circumference (all *p* < 0.001), indicating a consistent pattern of central adiposity. In contrast, psoriasis severity, as measured by PASI, was comparable between groups (*p* = 0.3), suggesting that excess body weight was not associated with greater clinical severity in this cohort. Metabolic parameters demonstrated a clear pattern of dysregulation among patients with overweight/obesity. These individuals had significantly higher fasting glucose and HbA1c levels (*p* = 0.002 and *p* < 0.001, respectively), indicating impaired glycemic control. Lipid profiles were also less favorable, with higher triglyceride and LDL-C levels and lower HDL-C concentrations (all *p* < 0.05). Total cholesterol did not differ significantly between groups (*p* = 0.7), suggesting that specific lipid fractions, rather than overall cholesterol levels, were more strongly associated with BMI status. In contrast, inflammatory markers, including TNF-α, IL-6, and IL-10, did not differ significantly between BMI groups (all *p* > 0.4). Given the cross-sectional design, sample size, and known biological variability of circulating cytokine measurements, this finding should not be interpreted as evidence against a role for systemic inflammation in obesity-related psoriasis pathophysiology.

Multivariable logistic regression results for overweight/obesity are presented in Table 2, with univariable estimates provided in Supplementary Table S3. Multicollinearity diagnostics did not indicate problematic collinearity among predictors in the final multivariable model (VIF range 1.09–1.50; Supplementary Table S4). Among metabolic parameters, higher triglyceride levels (log-transformed) were strongly associated with increased odds of overweight/obesity (adjusted OR 4.73, 95% CI 1.36–18.57, *p* = 0.019), indicating a robust relationship between dyslipidemia and excess body weight. Similarly, higher HbA1c levels were significantly associated with increased odds of overweight/obesity (adjusted OR 1.71, 95% CI 1.09–2.91, *p* = 0.031). In contrast, higher HDL-C levels were associated with significantly lower odds of overweight/obesity (adjusted OR 0.36, 95% CI 0.18–0.66, *p* = 0.003), suggesting a protective lipid profile. No statistically significant associations were observed for PASI, fasting glucose, total cholesterol, LDL-C and inflammatory biomarkers after multivariable adjustment (all *p* > 0.05), indicating that their effects were attenuated when considered alongside other metabolic factors. Because the study sample was frequency-matched 1:1 on overweight/obesity status, the adjusted odds ratios reported above describe the strength of association between each variable and overweight/obesity within this matched sample. They should not be interpreted as estimates of absolute risk, incidence, or population prevalence of overweight/obesity among patients with psoriasis.

The genotype distributions of the analyzed SNPs according to overweight/obesity status are presented in Table 3. A highly significant difference in genotype distribution was observed for rs10968110 in the *ZTV* gene (χ^2^ = 21.54, *p* < 0.001), indicating a significant association between this polymorphism and overweight/obesity. For rs10968110, the minor C allele was associated with higher odds of overweight/obesity compared with the T allele (allelic OR = 3.89, 95% CI: 2.14–7.06). The normal homozygous genotype TT was more frequent in the control group than in the overweight/obese group (78.7 ± 5.0% vs. 40.0 ± 5.7%). In contrast, the heterozygous genotype CT was more common among overweight/obese participants than controls (50.6 ± 5.8% vs. 18.7 ± 4.6%). The unfavorable homozygous genotype CC was also more frequent in the overweight/obese group (9.3 ± 2.9% vs. 2.7 ± 2.6%), further supporting the association of rs10968110 with overweight/obesity status. For rs1558902, the minor A allele was associated with higher odds of overweight/obesity compared with the T allele (allelic OR = 2.25, 95% CI: 1.37–3.71). Individuals carrying this polymorphism had more than twofold higher odds of overweight/obesity (OR = 2.25, 95% CI: 1.37–3.71). The normal homozygous genotype TT was more common in the control group than in the overweight/obese group (56.0 ± 5.8% vs. 30.6 ± 5.7%). The heterozygous genotype AT was more frequent among overweight/obese participants (57.3 ± 5.8% vs. 41.3 ± 5.8%), while the unfavorable homozygous genotype AA was also more prevalent in the overweight/obese group than in controls (12.0 ± 3.4% vs. 2.7 ± 2.6%). In contrast, no statistically significant difference in genotype distribution was observed for rs696574 in the *CALCRL* gene (χ^2^ = 1.33, *p* = 0.25). For rs696574, the tested allele was not significantly associated with overweight/obesity (OR = 0.72, 95% CI: 0.40–1.26).

The associations between candidate polymorphisms and overweight/obesity under multiple genetic models are presented in Table 4. For rs10968110, significant associations with BMI category were observed in the genotype model (GENO: χ^2^ = 23.30, *p* < 0.001), additive trend model (TREND: χ^2^ = 20.89, *p* < 0.001), allelic model (ALLELIC: χ^2^ = 21.54, *p* < 0.001), and dominant model (DOM: χ^2^ = 23.34, *p* < 0.001). In contrast, the recessive model was not statistically significant (REC: χ^2^ = 2.89, *p* = 0.086). These findings suggest that the association with overweight/obesity was primarily driven by the presence of at least one C allele rather than by homozygosity for the C allele alone. For rs1558902 in the *FTO* gene, statistically significant associations with overweight/obesity were observed across the genotype model (GENO: χ^2^ = 11.95, *p* = 0.003), additive trend model (TREND: χ^2^ = 11.95, *p* < 0.001), allelic model (ALLELIC: χ^2^ = 10.36, *p* = 0.001), and dominant model (DOM: χ^2^ = 9.80, *p* = 0.002). The recessive model was also significant using Pearson’s chi-square test (REC: χ^2^ = 4.81, *p* = 0.028), although this finding should be interpreted cautiously because of the small number of AA homozygotes. Overall, these results suggest that the A allele of rs1558902 is associated with higher odds of overweight/obesity, with the strongest support observed under additive, allelic, and dominant assumptions. In contrast, rs696574 in the *CALCRL* gene did not demonstrate statistically significant associations with BMI category in the additive trend, allelic, dominant, or recessive models. Therefore, the observed genotype and allele frequency differences for rs696574 do not provide clear evidence of association with overweight/obesity in the present sample.

To further investigate the combined genetic contribution of the analyzed polymorphisms to overweight/obesity, exploratory multifactor dimensionality reduction analysis was used to assess potential multilocus genotype interactions among the three candidate polymorphisms (Table 5). The single-locus model including rs10968110 demonstrated the highest cross-validation consistency among the evaluated models, with 10/10 consistency and a mean balanced accuracy of 0.654. The two-locus model combining rs1558902 (FTO) and rs10968110 showed modestly higher mean balanced accuracy (0.694), suggesting improved discriminatory performance compared with the single-locus model. However, its cross-validation consistency was lower (6/10), indicating reduced model stability. The three-locus model including rs1558902 (*FTO*), rs696574 (*CALCRL*), and rs10968110 did not further improve model performance, with a mean balanced accuracy of 0.692 and cross-validation consistency of 5/10. These findings suggest that rs10968110 was the most stable individual genetic predictor in the MDR framework, while the addition of rs1558902 (FTO) may provide limited complementary information. In contrast, rs696574 (CALCRL) did not appear to add meaningful discriminatory value in this sample. Given the relatively small sample size and reduced cross-validation consistency of the higher-order models, these findings should be interpreted cautiously and considered exploratory. Larger independent cohorts are needed to confirm whether these polymorphisms have reproducible multilocus interaction effects on overweight/obesity risk among patients with psoriasis.

## 4. Discussion

In this cross-sectional study of patients with psoriasis, we identified metabolic, glycemic, and candidate genetic correlates of overweight/obesity. The principal findings were threefold. First, overweight/obesity was independently associated with an adverse cardiometabolic profile, including higher triglyceride levels, higher HbA1c, and lower HDL-C levels. Second, circulating inflammatory markers did not differ significantly between BMI groups and were not independently associated with overweight/obesity after multivariable adjustment. Third, among the candidate genetic variants examined, rs10968110 (*ZTV*) and rs1558902 (*FTO*) showed evidence of association with overweight/obesity, while exploratory MDR analysis suggested that rs10968110 (*ZTV*) had the most stable discriminatory performance and that rs1558902 (*FTO*) may provide limited complementary information.

The observed metabolic profile is consistent with the established link between psoriasis and cardiometabolic comorbidity. Previous studies have shown that patients with psoriasis frequently exhibit features of metabolic syndrome, including dyslipidemia characterized by elevated triglycerides and reduced HDL-C [18,19,20]. Our findings align with this literature, suggesting that lipid abnormalities may represent a central pathway linking psoriasis and excess adiposity. The lack of association between BMI and PASI scores in our cohort is also in line with several reports indicating that psoriasis severity is not always directly correlated with body mass index, particularly in heterogeneous clinical populations [21,22,23].

Beyond the metabolic and genetic associations reported here, a broader dermatological literature supports a bidirectional and multidimensional relationship between obesity and psoriasis. Obesity has repeatedly been identified as a modifier of both psoriasis severity and treatment response: excess body weight is associated with higher PASI scores and reduced response to biologic and phototherapy in several cohorts [24,25], although this pattern was not observed for PASI in our own cohort, possibly reflecting differences in population, treatment exposure, or disease duration that were not captured in the present dataset. Obesity is also a core component of metabolic syndrome, which is substantially more prevalent among patients with psoriasis than in the general population and is itself linked to greater disease severity and cardiovascular risk [26]; the pattern of elevated triglycerides, higher HbA1c, and reduced HDL-C observed in our overweight/obese group is consistent with a metabolic-syndrome-like phenotype and reinforces the case for integrated cardiometabolic screening in psoriasis care. Mechanistically, adipose-tissue-derived mediators such as adiponectin and leptin, together with markers of gut-derived and oxidative metabolic stress such as trimethylamine-N-oxide (TMAO) and oxidized LDL, have been proposed as pathways linking obesity to psoriasis pathogenesis beyond classical circulating cytokines [27,28,29]. These mediators were not measured in the present study and represent a natural extension of this work. Finally, ethnicity-related differences in body composition and metabolic risk thresholds, reflected here in our use of an Asian-specific BMI cutoff [14,15,16], suggest that the metabolic correlates of overweight/obesity identified in this ethnic Kazakh cohort may not directly generalize to populations classified using standard WHO BMI categories, underscoring the value of ethnicity-specific studies of this kind.

Notably, circulating TNF-α, IL-6, and IL-10 levels were not significantly different between BMI groups in this sample. This negative finding should be interpreted cautiously and should not be taken to indicate that systemic inflammation is unrelated to obesity in psoriasis; rather, it may reflect limited statistical power, biological variability in cytokine measurement, and the cross-sectional design, as detailed below. Although psoriasis is regarded as a systemic inflammatory disease and obesity is associated with chronic low-grade inflammation, the absence of detectable differences in these cytokines may reflect several factors. First, circulating cytokine levels may not fully capture local tissue-level inflammation relevant to psoriasis pathophysiology [7,30]. Second, variability in disease duration, treatment status, comorbidities, and individual immune responses may have attenuated measurable between-group differences. Third, the cross-sectional design limits the ability to assess dynamic inflammatory processes over time. These findings are consistent with studies reporting inconsistent associations between systemic cytokine levels and obesity in psoriasis [30,31].

From a genetic perspective, our findings provide evidence for a dual contribution of both rs10968110 (*ZTV*) and rs1558902 (*FTO*) to overweight/obesity risk. The *ZTV* variant demonstrated the strongest and most stable association, with significant findings across genotype, additive trend, allelic, and dominant models. For rs1558902 in FTO, the association was most evident in the additive trend and allelic models, suggesting that the A allele may be associated with higher odds of overweight/obesity in a dose-related manner. This pattern is biologically plausible and consistent with prior literature showing that *FTO* variants are associated with modest but cumulative differences in adiposity and obesity risk [11,13]. Furthermore, previous studies have reported that rs1558902 in the *FTO* gene is associated with higher BMI specifically in patients with type I psoriasis, suggesting potential heterogeneity by disease subtype [13].

It is also important to note that the three candidate SNPs evaluated here were originally identified through genome-wide or candidate-gene studies conducted predominantly in European, East Asian, or other non-Central-Asian populations. Detailed characterization of the baseline genetic background of the Kazakh population, including genome-wide allele-frequency spectra, population structure, and linkage disequilibrium patterns, remains limited. We therefore cannot exclude the possibility that population-specific genetic architecture distinct from that of the discovery populations influenced the observed associations or their generalizability. The allele frequencies observed for rs1558902, rs696574, and rs10968110 in the present Kazakh sample (Table 3; Supplementary Table S1) provide a preliminary reference point, but larger population-genetic studies specifically characterizing Central Asian and Kazakh ancestry groups are needed to contextualize these findings and to identify additional, potentially population-specific, obesity-susceptibility variants.

The model-specific pattern observed for rs1558902 is consistent with the polygenic architecture of obesity, in which individual variants often show modest effects that are more detectable under additive or allelic assumptions. *FTO* variants have been linked to energy balance, appetite regulation, and adiposity, providing biological plausibility for their association with elevated BMI [32,33]. The weaker findings under some genetic models should be interpreted cautiously because the sample size was limited and genotype subgroup counts were small. The two-locus MDR model including rs1558902 and rs10968110 showed modestly higher balanced accuracy than the single-locus model, but its lower cross-validation consistency indicates reduced stability; therefore, this finding should be viewed as exploratory rather than confirmatory evidence of interaction.

While the present study did not measure adipokines or metabolites such as TMAO or oxidized lipids, these mediators have been proposed to mechanistically link obesity and psoriasis and may interact with the metabolic pathways implicated by the FTO and ZTV variants examined here [27]. Incorporating such measures in future extensions of this cohort could help clarify whether the genetic associations identified in this study operate through adipokine-mediated inflammatory pathways, oxidative-lipid pathways, or largely independent mechanisms.

From a clinical and public health perspective, these findings have several implications. First, the associations of triglycerides, HDL-C, and HbA1c with overweight/obesity underscore the importance of integrating cardiometabolic screening into routine psoriasis care. Early identification and management of dyslipidemia and impaired glycemic control may help reduce long-term cardiometabolic and cardiovascular risk in this ethnic Kazakh population. Second, the observed associations for rs10968110 and rs1558902 may inform future research on genetic susceptibility to overweight/obesity in psoriasis, although these markers are not ready for clinical risk stratification without replication in larger and independent cohorts. Finally, the lack of association between BMI category and the measured inflammatory biomarkers suggests that metabolic risk assessment in psoriasis should include direct evaluation of lipid and glycemic profiles rather than relying on circulating cytokine levels alone.

This study has several limitations. The cross-sectional design precludes causal inference and limits the ability to assess temporal relationships between metabolic, inflammatory, and genetic factors. The sample size was modest, particularly for genetic subgroup and MDR analyses, which may have limited power to detect smaller effects and reduced the stability of higher-order interaction models. Data on systemic psoriasis treatment, disease duration, smoking status, alcohol consumption, physical activity, and dietary habits were not collected in this study and could therefore not be adjusted for in the regression models. These unmeasured factors are plausible sources of residual confounding for both metabolic parameters and BMI status, and their absence should be taken into account when interpreting the reported associations. Prospective collection of these variables is recommended for future studies in this population. In addition, lack of stratification by psoriasis subtype and limited information on population genetic structure may have obscured subtype- or ancestry-specific associations. In addition, because participants were sampled using a fixed 1:1 frequency-matched design rather than as a representative cross-sectional sample, the odds ratios reported throughout this study reflect within-sample associations and should not be extrapolated as estimates of overweight/obesity risk or prevalence in the broader psoriasis population. The candidate-gene design and exploratory MDR analysis also require cautious interpretation and external replication.

## 5. Conclusions

In conclusion, overweight/obesity in ethnic Kazakh patients with psoriasis was associated with an adverse cardiometabolic profile, particularly elevated triglycerides, higher HbA1c, and reduced HDL-C, while measured circulating inflammatory markers were not independently associated with BMI category. The candidate genetic findings suggest that rs10968110 (*ZTV*) and rs1558902 (*FTO*) may be associated with overweight/obesity susceptibility, but these results should be considered exploratory until validated in larger independent cohorts. These findings highlight the importance of integrated cardiometabolic assessment in psoriasis management and support further investigation of genetic markers as potential research tools for understanding obesity susceptibility in this population.

## Figures and Tables

**Table 1:** Baseline characteristics of psoriasis patients according to overweight/obesity status.

Characteristic	Overall N = 150 (% or SD)	Overweight/Obese N = 75 (% or SD)	Control N = 75 (% or SD)	*p*-Value
Age	49.83 (16.66)	51.59 (13.42)	48.87 (18.45)	0.3
Gender				0.2
Female	83 (55%)	46 (61%)	37 (49%)	
Male	67 (45%)	29 (39%)	38 (51%)	
Height	166.95 (8.98)	166.16 (9.00)	167.75 (8.95)	0.4
Weight	81.36 (17.64)	94.20 (13.98)	68.51 (9.84)	<0.001
BMI	29.25 (6.07)	34.15 (4.29)	24.36 (2.67)	<0.001
Waist	98.03 (14.35)	109.32 (9.73)	86.73 (7.83)	<0.001
PASI	15.18 (8.28)	15.46 (8.26)	14.90 (8.34)	0.3
Glucose	5.76 (2.36)	6.29 (2.91)	5.24 (1.49)	0.002
Hb1Ac	6.14 (1.57)	6.54 (1.78)	5.74 (1.23)	<0.001
Cholesterol	5.01 (1.00)	5.04 (0.97)	4.97 (1.04)	0.7
Triglyceride	1.83 (1.24)	2.10 (1.17)	1.55 (1.26)	<0.001
LDL-C	3.26 (0.99)	3.43 (1.02)	3.09 (0.92)	0.024
HDL-C	1.58 (0.81)	1.42 (0.68)	1.73 (0.90)	0.007
TNF-α	2.94 (3.72)	2.53 (2.35)	3.35 (4.70)	0.8
IL-6	2.53 (2.32)	2.81 (2.56)	2.26 (2.02)	0.4
IL-10	3.44 (4.65)	4.28 (6.02)	2.60 (2.42)	>0.9

Abbreviations: BMI, body mass index; HbA1c, glycated hemoglobin; HDL-C, high-density lipoprotein cholesterol; IL-6, interleukin-6; IL-10, interleukin-10; LDL-C, low-density lipoprotein cholesterol; N, number of participants; PASI, Psoriasis Area and Severity Index; SD, standard deviation; TNF-α, tumor necrosis factor-alpha.

**Table 2:** Multivariable logistic regression model for overweight/obesity among patients with psoriasis.

Variable	Adjusted OR (95% CI) ^1^	*p*-Value
PASI	1.01 (0.96–1.06)	0.668
Fasting glucose	1.06 (0.78–1.49)	0.718
HbA1c	1.71 (1.09–2.91)	0.031
Total cholesterol	0.75 (0.43–1.27)	0.299
Triglycerides, log-transformed	4.73 (1.36–18.57)	0.019
LDL-C	1.34 (0.78–2.37)	0.302
HDL-C	0.36 (0.18–0.66)	0.003
TNF-α, log-transformed	0.86 (0.44–1.63)	0.649
IL-6, log-transformed	1.33 (0.64–2.77)	0.442
IL-10, log-transformed	0.81 (0.47–1.42)	0.463

^1^ Note. The model was additionally adjusted for age and sex. Abbreviations: HbA1c, glycated hemoglobin; HDL-C, high-density lipoprotein cholesterol; IL-6, interleukin-6; IL-10, interleukin-10; LDL-C, low-density lipoprotein cholesterol; PASI, Psoriasis Area and Severity Index; TNF-α, tumor necrosis factor-alpha.

**Table 3:** Genotype and allele frequency distributions of candidate polymorphisms according to overweight/obesity status.

SNP/Gene	Genotype or Allele	Overweight/Obese, N (%)	Control, N (%)	χ^2^	*p*-Value	OR (95% CI)
rs696574/*CALCRL*	Genotype model			3.04	0.218	—
	CC	51 (68.0)	45 (60.0)			
	CT	22 (29.3)	26 (34.7)			
	TT	2 (2.7)	4 (5.3)			
	Allelic model			1.33	0.248	0.72 (0.40–1.26)
	T allele	26 (17.3)	34 (22.7)			
	C allele	124 (82.7)	116 (77.3)			
rs10968110/*ZTV*	Genotype model			23.30	<0.001	—
	TT	30 (40.0)	59 (78.7)			
	CT	38 (50.7)	14 (18.7)			
	CC	7 (9.3)	2 (2.7)			
	Allelic model			21.54	<0.001	3.89 (2.14–7.06)
	C allele	52 (34.7)	18 (12.0)			
	T allele	98 (65.3)	132 (88.0)			
rs1558902/*FTO*	Genotype model			11.95	0.003	—
	TT	23 (30.7)	42 (56.0)			
	AT	43 (57.3)	31 (41.3)			
	AA	9 (12.0)	2 (2.7)			
	Allelic model			10.36	0.001	2.25 (1.37–3.71)
	A allele	61 (40.7)	35 (23.3)			
	T allele	89 (59.3)	115 (76.7)			

Note. Genotype percentages were calculated using the number of participants in each group as the denominator (n = 75 per group). Allele frequencies were calculated using the total number of alleles as the denominator (2n = 150 per group). For genotype rows, χ^2^ and *p*-values correspond to the genotype model. OR = odds ratio; CI = confidence interval; SNP = single nucleotide polymorphism; χ^2^ = chi-square statistic. OR—crude allelic ORs comparing A1 vs A2. Abbreviations: SNP—Single nucleotide polymorphism; BMI—Body mass index; χ^2^—Chi-square statistic.

**Table 4:** Associations between candidate polymorphisms and overweight/obesity (BMI > 28 kg/m^2^) under multiple genetic models.

Gene	SNP	A1	A2	Model	Overweight/Obese	Control	χ^2^	DF	*p*-Value
*CALCRL*	rs696574	T	C	GENO	2/22/51	4/26/45	3.04	2	0.218
		T	C	TREND	26/124	34/116	1.33	1	0.248
		T	C	ALLELIC	26/124	34/116	1.33	1	0.248
		T	C	DOM	24/51	30/45	2.89	1	0.089
		T	C	REC	2/73	4/71	0.69	1	0.405
*ZTV*	rs10968110	C	T	GENO	7/38/30	2/14/59	23.30	2	<0.001
		C	T	TREND	52/98	18/132	20.89	1	<0.001
		C	T	ALLELIC	52/98	18/132	21.54	1	<0.001
		C	T	DOM	45/30	16/59	23.34	1	<0.001
		C	T	REC	7/68	2/73	2.89	1	0.089
*FTO*	rs1558902	A	T	GENO	9/43/23	2/31/42	11.95	2	0.0025
		A	T	TREND	61/89	35/115	11.95	1	<0.001
		A	T	ALLELIC	61/89	35/115	10.36	1	0.001
		A	T	DOM	52/23	33/42	9.80	1	0.001
		A	T	REC	9/66	2/73	4.81	1	0.028

Note. χ^2^= Pearson chi-square statistic, except TREND, which uses the Cochran–Armitage trend test. Abbreviations: SNP—Single nucleotide polymorphism; GENO—Genotype model (3 × 2 comparison); TREND—Cochran–Armitage trend (additive) model; ALLELIC—Allelic model (2 × 2 allele comparison); DOM—Dominant model (carrier vs. non-carrier of A1); REC—Recessive model (A1 homozygotes vs. others); χ^2^—Chi-square statistic; DF—Degrees of freedom; *p*—*p*-value.

**Table 5:** Exploratory multifactor dimensionality reduction analysis of combined rs1558902 (FTO), rs696574 (CALCRL), and rs10968110 polymorphisms in relation to overweight/obesity among patients with psoriasis.

Model	Order	CV Consistency	Mean Sensitivity	Mean Specificity	Mean Accuracy	Mean Balanced Accuracy
rs10968110 *ZTV*	1	10/10	0.561	0.748	0.654	0.654
rs1558902 *FTO* + rs10968110 *ZTV*	2	6/10	0.614	0.773	0.694	0.694
rs1558902 *FTO* + rs696574 *CALCRL* + rs10968110 *ZTV*	3	5/10	0.600	0.784	0.692	0.692

## Data Availability

The original contributions presented in this study are included in the article/Supplementary Materials. Further inquiries can be directed to the corresponding author.

## Supplementary Materials

The following supporting information can be downloaded at: https://www.mdpi.com/article/10.3390/medicina62081448/s1. Table S1: Hardy–Weinberg equilibrium test results for the analyzed polymorphisms; Table S2: Data analyzed in the present study; Table S3: Univariable logistic regression of factors associated with overweight/obesity; Table S4: Multicollinearity assessment results.
