# Supplementary material for: Metabolic and Candidate Genetic Factors Associated with Overweight/Obesity in Patients of Kazakh Ethnicity with Psoriasis: A Cross-Sectional Study
Source: Medicina (Kaunas). 2026 Jul 26;62(8):1448. doi: 10.3390/medicina62081448 (PMC13514493; doi:10.3390/medicina62081448)
Supplement: Supplementary file 1 [file medicina-62-01448-s001.zip › medicina-4388336-supplementary.pdf]

Table S1. Hardy–Weinberg equilibrium test results for the analyzed polymorphisms

Gen	SNP	TEST	A1	A2	GENO	O(HET)	E(HET)	χ^2	DF	P
CALCRL	rs696574	ALL	T	C	6/48/96	0.3200	0.3200	0.0000	1	1.0000
ZTV	rs10968110	ALL	C	T	9/52/89	0.3467	0.3578	0.1447	1	0.6535
FTO	rs1558902	ALL	A	T	11/74/65	0.4933	0.4352	2.6765	1	0.1336

Abbreviations: Gen – Gene; SNP – Single nucleotide polymorphism; TEST – Test performed (ALL = overall sample); A1 – Effect (tested) allele; A2 – Alternative allele; GENO – Genotype counts (A1A1/A1A2/A2A2); O(HET) – Observed heterozygosity; E(HET) – Expected heterozygosity under Hardy–Weinberg equilibrium; χ^2 – Chi-square statistic; DF – Degrees of freedom; P – P-value

Table S2. Data analyzed in the present study.

Age	Gender	Height	Weight	BMI	Waist Cir	PASI	Cholesterol	Triglyceride	LDLP	HDLP	Glucose	Hb1a	Fno-Alpha	Il-6	Il-10
19	2	160	42	16.40	62	27.7	3.63	1.62	1.98	1.29	3.87	5.3	1	0.47	1.89
19	1	187	66	18.90	74	12	6.84	0.88	3.85	2.7	5.14	5.5	14.6	2.42	6
48	2	170	56	19.40	77	10	4.87	0.71	2.71	1.4	3.91	5.56	1	0.42	0.98
18	1	178	63	19.90	77	26.9	4.66	1.85	2.4	1.14	3.33	5.7	3.68	8.1	4.25
33	1	175	62	20.20	79	27	2.9	2.74	1.54	1.01	4.2	5	0	1.99	8
37	1	172	60	20.30	83	29.6	5.87	3.71	4.71	2.3	8.91	6.56	1.48	2.09	1.88
47	2	163	54	20.30	84	31.9	5.6	1.09	3.54	2.01	4.84	4.9	3	4.8	3
21	1	180	66	20.40	77	16.2	4.12	0.54	2.84	0.87	4.38	5.6	1.96	1.12	1.64
25	1	175	64	20.90	73	10.4	3.46	0.33	1.17	1.54	5.29	5	3.78	0.59	7
20	1	183	71	21.20	75	13.8	3.38	0.58	1.66	1.24	4.12	5.41	0	1.82	0.98
18	1	188	75	21.20	80	24	5.32	4.3	2.23	4.65	5.71	5.9	2	1	5.94
34	1	185	73	21.30	90	19	5.76	0.72	1.97	1.49	4.3	5.15	6.24	1.3	0.41
42	2	165	58	21.30	85	10	5.1	1	2.5	2.2	5.7	5.3	20.41	1.21	1.15
32	2	156	52	21.40	77	5.4	5.89	1.82	2.49	2.17	4.5	5.72	1	0.95	0.82
19	1	180	70	21.60	86	10	4.86	1.79	2.14	2.79	4.6	6.1	2	3.58	2.3
39	2	157	54	21.90	85	13.8	4.72	1.6	3.59	1.35	6	5.15	7.15	0.29	1.31
37	2	164	60	22.30	80	10.2	4.83	0.85	3.15	1.34	5.2	4.7	3.87	8.35	6
57	2	161	58	22.40	85	12.6	6.53	2.3	4.13	5.33	4.58	6	11	1.58	2.55
70	2	152	52	22.50	83	26.1	4.5	0.95	2.24	1.48	6.1	6.01	1.15	2.35	0.25
38	1	168	63.8	22.60	80	8.5	4.45	0.99	3.67	0.98	4.32	5.23	1	1.7	0
22	2	158	57	22.80	80	15	3.8	0.59	2.47	1.98	5.4	5.7	0	4.2	3
73	2	158	57	22.80	91	14.6	6.2	3.3	2.13	5.33	5.3	4.9	5.07	1.04	0.08
74	1	164	62	23.10	84	10	5.54	0.6328	3.31	1.2	8.74	8.7	0.9	3.64	14
25	2	165	63	23.10	75	10.8	4.78	1.38	3.14	1.42	4.81	4.3	1	8.25	5
66	1	164	62	23.10	85	12	6.13	0.98	3.94	1.7	4.82	5.6	2.23	2.4	4.36

69	2	167	65	23.30	85	8.2	6.65	0.84	4.07	2.09	4.58	5.7	1	1.34	1.07
37	1	173	70	23.40	85	31.5	4.76	0.59	3.54	1.35	6.19	5.8	1.8	3.51	1.6
49	1	180	76	23.50	90	5.9	5	2.5	3.2	1.4	5.2	5.5	1	4.29	2.26
67	2	161	61	23.50	95	11.4	6.1	1.5	5.25	1.73	12	9.6	4.86	3.54	2.95
20	2	162	62	23.60	77	10	4.97	1.25	2.76	1.19	5.7	6.13	1	1.5	1.45
27	2	157	59	23.90	79	10.8	2.72	0.61	1.28	1.09	4.86	5.1	0.82	1	4.52
52	1	165	65	23.90	90	10	4.5	0.6	3.31	0.919	4.9	5.8	4.46	1.78	4.02
40	1	175	75	24.50	103	13	4.8	0.85	3.4	1.34	2.2	4.7	1	3.27	1.48
67	1	172	75	24.50	94	33.4	5.2	2.4	3.1	1.9	5	6.35	1	6.35	4.18
41	1	175	75	24.50	96	13.4	5.2	3	3.7	1.9	4.6	5.73	1.28	0.03	0
31	2	154	58	24.50	87	10.1	4.75	0.62	2.7	1.65	5.03	5.3	3.74	0.65	2.98
50	2	165	67	24.60	97	29	3.76	1.95	3.17	1.48	4.87	6.71	1	2.48	1.31
45	1	170	71	24.60	88	12.1	5.6	0.81	3.11	1.41	4.58	6	2.87	1.79	7.02
67	1	167	69	24.70	95	12	5.72	1.18	4.22	1.29	3.3	5.3	1	1.04	2.21
54	2	166	68.5	24.90	85	17.1	5.42	1.17	2.37	2.35	5.37	3.33	1	0.95	0.33
21	2	150	56	24.90	83	6.5	4.09	1.03	2.49	1.4	5.28	5.4	1	0.34	4.1
45	1	170	72	24.90	88	30.6	3.98	0.85	2.79	2.34	5.19	5.7	6	2.51	4
67	2	155	60	25.00	90	3.6	4.6	2.56	2.7	1.1	4.5	5.95	1.42	1.74	2.95
65	1	167	70	25.10	85	18	5.35	0.93	2.2	1.1	5.4	5.32	1	1.08	2.38
69	2	152	58	25.10	80	10	5.2	1.2	3.3	1.8	5.7	5.4	2.64	2.18	1.89
27	1	173	75	25.10	93	14.4	4.84	1.56	3.7	0.93	6.1	5.26	3.85	4.9	1.15
41	1	169	73	25.60	92	17.3	4.37	0.85	3.01	1.18	4.6	5.1	17.18	0.64	0.41
53	2	167	72	25.80	82	10	5.89	0.3	3.13	1.98	4.99	6.23	1	1.08	0.57
71	1	172	77	26.00	99	11	5.1	1.14	3.23	1.53	5.13	6.38	1	9.52	1.89
72	2	158	65	26.00	87	10.8	4.69	8.89	3.22	1.42	4.54	4.16	11.9	1.4	1.72
25	2	164	70	26.00	85	5.9	4.6	0.7	3.2	1.4	4.9	4.9	21.12	1.14	0
37	2	165	71	26.10	84	10.7	3.14	0.68	1.16	2.79	3.58	4.73	8	3.58	0.78
18	1	179	84	26.20	90	13.5	4.31	1.8	2.49	1.22	4.44	5.6	4.05	0.2	1.23
29	2	170	76	26.30	89	16	7.96	0.98	4.85	3.7	5.19	5.6	1.69	0.77	1.97
33	1	169	76	26.60	93	9	5.3	1.1	2.3	1.1	4.5	5.13	18.89	1.56	6.59
63	2	165	73	26.80	99	12	5.78	1.9	2.4	1.08	5.6	6.01	1	0.95	1.56
54	2	170	77.6	26.90	86	10.6	5.76	2.61	4.66	1.35	6.28	5.8	1	1.7	0.9
19	1	165	73.3	26.90	85	10	3.34	0.58	1.56	1.79	4.58	4.23	1	1.3	0
57	1	167	65	26.90	98	7.4	4.78	2.9	3.4	1.08	5.7	6.09	1	0.55	0.66
59	1	169	77	27.00	95	22.5	2.4	1.46	3.62	1.2	5.5	6.09	1	5.3	6.71
35	2	168	77	27.30	90	7.2	5.6	0.81	3.22	1.18	4.58	5.3	0.66	0.86	1.38
53	2	170	79	27.30	99	15.2	7.76	0.94	5.8	1.5	11.83	12.4	1.97	2.74	0.98
63	2	168	77	27.30	91	10	5.2	1.29	2.54	1.79	5.5	6.7	2	0.82	2.59
62	2	160	70	27.30	83	7.2	4.4	3.33	3.4	1.8	4.6	6	5.67	0.82	1.07
61	2	150	62	27.60	92	13.2	5.8	2.1	4.5	1.1	6.2	6.9	1	1.43	4.1
85	2	150	62	27.60	79	22.7	4.02	1.11	3.28	1.35	5	4	1.77	2.28	3.78

70	1	170	80	27.70	103	10	4.73	0.85	2.56	1.7	5.09	6.18	0.65	4.32	2.12
36	1	174	84	27.70	99	12.6	4.66	1.28	2.99	1.36	5.34	5.4	1	0.69	0.66
31	1	173	83	27.70	88	49	5	0.9	3.4	1.1	5	6.11	2	2.94	0.89
18	1	180	90	27.80	98	29.7	4.76	0.95	3.24	1.48	4.3	6.01	0	4.2	1.65
61	2	161	72	27.80	83	10	4.69	1.28	2.88	1.5	7.71	8.74	0	1.12	3
33	1	177	87	27.80	93	17	5.78	3.7	4.04	1.08	4.7	5.09	0.75	2.65	1.84
37	1	183	92	27.80	90	10	4.7	3.1	4.45	3.74	5.1	4.7	1	0.99	0.49
19	1	180	90	27.80	98	10.9	4.3	3.1	4.38	2.21	4.6	6	2	1	7.3
52	2	164	75	27.90	83	7.4	6.7	0.65	2.79	1.34	5.1	6	1.89	0.77	1.56
34	2	162	76	29.00	100	6	4.57	1.53	2.38	1.89	3.79	8.67	0.76	0	1
55	2	173	87	29.10	106	15.4	4.5	1.5	2.43	1.83	5	5.1	1.62	3.65	4.54
38	1	170	84	29.10	105	6	4.67	0.53	3.28	1.29	5.89	5.97	0.65	0.65	9
52	2	175	90	29.40	102	10	3.71	0.83	1.92	1.3	5.33	5.8	1	0.99	1.48
68	1	167	82	29.40	110	12.6	4.66	1.3	6.39	1.33	5.5	4.78	1	0.77	2.54
69	1	170	85	29.40	105	40	4.5	2.43	3.37	2.59	5.3	6.38	1.48	5	0
53	1	167	82	29.40	110	17.5	3.53	2.68	2.45	1.27	6.03	4.9	2.03	4.68	1.8
44	1	181	97	29.60	115	15.2	4.6	1.17	3.04	0.71	4.8	6.9	4.46	3.8	27.43
57	1	176	93	30.00	104	17.7	4.8	2.01	5.03	0.91	2.2	7.8	0	8	7
65	2	165	82	30.10	117	7	6.5	1.9	4.9	1.2	6	6.8	1	2.27	3.1
58	2	172	89	30.10	110	11	5.12	1.23	3.53	1.47	4.15	6.3	1	1.21	0
50	2	171	88	30.10	102	10	2.91	0.73	1.28	1.16	4.52	6.44	3	2.89	0.82
24	2	165	82	30.10	87	10.8	3.9	1.7	4.1	1	5.9	4.6	4.12	1.83	1.48
23	1	191	110	30.20	108	9	6.01	2.8	4.54	0.76	6.69	5.67	2.49	3.75	1.79
46	2	171	89.3	30.40	103	16	6.53	0.84	5.24	1.34	6.1	5.7	1	3.63	2.05
51	2	170	88	30.40	104	11.2	6.3	0.9	2.6	1.7	5.4	6.2	1.12	3.83	1.03
43	1	174	92	30.40	109	31.6	5.18	5.99	3.32	0.86	21.32	14.2	1.49	0.95	2.79
60	2	160	78	30.50	99	13	5.52	2.23	3.43	1.37	5.15	6.4	1	6.83	1.64
69	1	175	94	30.70	117	14.5	6.01	2	4.64	0.746	7.69	8.67	1	5.34	2.7
29	1	170	89	30.80	111	16.2	3.87	0.55	2.38	1.1	3.86	5.77	1	1.08	0.66
76	2	150	70	31.10	99	13.6	4.1	1.2	2.8	1.5	5.3	6.1	1	0.6	0.9
61	1	170	90	31.10	97	11	6.01	2.9	3.54	0.76	6.59	5.67	1	9.58	0.789
29	2	170	90	31.10	95	22.8	4.4	1.58	2.4	0.81	4.5	5.5	4.32	4.85	0.82
68	1	166	85	31.20	100	10	5.6	1.3	1.64	0.91	5.13	6.1	1	0.29	0.49
50	2	165	85	31.20	100	10.1	6.02	2.9	3.54	1.76	4.48	5.9	4.03	8.68	2.8
48	2	165	86	31.60	97	10.7	5.3	1.36	3.38	1.3	6.55	5	1	1.43	1.31
53	2	160	81	31.60	103	20	4.5	1.68	3.4	1.81	5	5.5	1.23	1.03	3.31
65	2	166	88	31.90	98	16	4.65	2.68	3.74	2.81	5.2	6.5	4.49	2.88	0
52	2	165	87	32.00	105	10	6.1	3.11	4.72	1.14	5.3	5.6	1.01	0.86	0.49
65	2	155	77	32.00	103	46.5	4.87	3.98	2.29	2.24	6.19	6.9	2.42	3.6	0
40	2	164	87	32.30	105	11.4	4.1	1.1	2.6	1.4	5.3	4.9	5	3.58	1.45
38	1	176	100	32.30	103	18	5.02	1.14	3.5	1.17	5.49	5	7	3	0

67	2	151	74	32.50	90	11	6.93	2.54	4.9	1.18	6.56	5.7	1	1.56	0
69	1	175	100	32.70	124	29	5.6	1.2	1.55	0.81	4.58	8.18	1.01	2.57	0
45	1	175	100	32.70	107	7	4.31	1.4	3.8	2.5	5.1	4.8	8.58	2.75	7.94
66	1	172	97	32.80	114	12	5.9	6	4.6	4.09	11.5	6.7	1.45	6.8	8.19
67	2	160	84	32.80	100	13	5.31	2.4	4.8	3.5	6.1	7.8	6.13	9.2	3
53	2	160	85	33.20	107	10	4.61	1.88	3.19	1.04	3.5	5.8	1	0.29	0
63	1	164	90	33.50	117	18.9	4.3	0.82	2.23	0.89	4.5	6.1	3.65	2.48	1.39
63	1	185	115	33.60	125	11.2	5.47	1.88	3.6	0.99	6.98	6.3	1	0.73	0.33
39	2	169	96	33.60	99	10.2	5.32	1.57	3.06	1.64	5.21	5.73	1	1.3	1.64
48	2	165	92	33.80	118	11.2	4	1.03	3.29	0.98	5	5.67	1.95	0	3.51
59	1	165	92	33.80	110	17	6.32	1.54	4.35	2.32	4.16	8.7	6	7.73	4
53	1	180	110	34.00	105	19.9	5.1	1.56	3.46	0.83	7.42	5.9	7.07	1.47	9.41
38	2	166	94	34.10	102	16.2	3.84	2.23	3.33	1.03	6.02	5.9	1	1.72	1.08
74	1	170	100	34.60	124	13.5	6.41	3.44	3.65	2.32	9.09	6.9	2.06	2.34	11.07
57	2	150	78	34.70	108	19.8	5.85	1.89	4.39	1.09	6.95	6.2	1	1.56	0.41
50	1	178	110	34.70	117	10	3.88	1.71	2.78	0.76	5.41	6.2	3	7.93	10.9
63	2	143	71	34.70	115	15	5.1	2.64	3.46	0.83	7.42	5.9	3	3.84	9.1
54	2	159	88	34.80	115	13.4	5.94	2.89	3.39	2.09	14.5	8.9	3.1	5.86	15
41	2	157	86	34.90	119	45	4.77	1.98	3.29	1.24	7.19	5.9	1	0.34	1.07
62	2	150	79	35.10	103	16	4.68	1.09	3.77	1.34	4.41	5.3	1	0.23	1.23
66	2	162	92	35.10	114	18	5.32	2.54	4.35	1.32	6.16	6.7	8	4.59	9
56	1	176	109	35.20	126	17	4.5	4.7	3.47	0.804	16.2	13	2.84	1.21	0.74
65	2	160	90	35.20	118	11	3.35	0.76	1.7	1.71	5.81	6.34	3.58	0.73	0
54	1	162	88	35.50	104	8	6.9	1.2	4.5	1.8	5.4	4.7	1	1	0.49
36	2	164	97	36.10	112	17.1	5.51	3.19	3.58	0.89	4.61	6.19	1	1.83	0
47	2	162	95	36.20	102	14.4	6.31	1.44	4.65	1.32	11.09	7.9	1	0.47	0.41
47	1	178	119	37.60	103	24	3.4	0.9	1.5	0.8	4.32	4.7	5.23	9.1	29.46
45	1	174	115	38.00	119	10.8	6.3	2	4.3	2	6.2	6.5	2.49	5.7	11.2
76	2	150	86	38.20	104	12.6	6.4	1.8	4	2.4	5.6	6.2	1	0.6	0.41
62	2	153	90	38.40	98	11.7	4	4.3	2.4	1.6	5	6.3	1	0.25	13.5
52	2	150	88	39.10	117	10	5.6	4.32	5.37	2.51	7.34	10.8	2	3.19	6.78
42	2	162	103	39.20	117	17.1	4.6	4.02	3.37	1.51	10.34	12.38	1	0.29	1.23
27	2	160	101	39.50	107	10.8	3.67	1.2	2.32	1.4	4.5	5.8	1	1.04	1.72
24	1	175	121	39.50	110	10	4.31	2.11	3.03	0.75	4.61	5.5	1	0.73	0
58	2	163	105	39.50	107	21.3	6.24	0.94	3.1	2.5	5.97	5.5	2	1.92	2.82
57	2	168	113	40.00	107	8.6	3.61	1.2	2.18	1.24	4.58	5.57	4.91	0.73	8
64	2	150	91	40.40	122	6	5.81	3.4	4.62	1.34	4.28	6.4	1	1.12	0.66
49	2	158	103	41.30	127	15	5.71	3.3	4.52	1.44	5.28	6.4	11	0.25	14
76	2	172	125	42.30	130	10.4	5.71	3.23	3.32	0.34	5.8	7.4	7.22	5.85	3.9
23	1	169	122	42.70	119	11	5.81	3.2	3.62	0.34	5.28	6.4	9	4.55	19
42	1	170	130	45.00	128	23.5	3.64	2.66	2.5	0.71	9	5.8	1	1.61	7.39

56	2	168	130	46.10	130	21.4	5.6	1.3	4.1	1.5	12	8.4	1	0.42	0
56	2	165	128	47.00	130	40	4.3	2.3	2.37	1.59	5.3	5.38	1	1.23	16

Table S3. Univariable logistic regression of factors associated with overweight/obesity

Term	Crude OR (95% CI)	p-value
Age	1.03 (0.87, 1.06)	0.213
Gender (Ref. Female)		
Male	0.61 (0.32, 1.17)	0.140
PASI	1.01 (0.97, 1.05)	0.675
Glucose	1.30 (1.08, 1.64)	0.013
Hb1A	1.55 (1.18, 2.17)	0.005
Cholesterol	1.08 (0.78, 1.50)	0.633
Triglyceride (log)	2.97 (1.67, 5.52)	<0.001
LDL-C	1.45 (1.04, 2.06)	0.034
HDL-C	0.59 (0.35, 0.91)	0.027
IL 6 (log)	1.02 (0.77, 1.36)	0.891
IL 10 (log)	0.92 (0.78, 1.09)	0.346
TNF alpha (log)	1.10 (0.86, 1.42)	0.459

Abbreviations: CI, confidence interval; HbA1c, glycated hemoglobin; HDL-C, high-density lipoprotein cholesterol; IL-6, interleukin-6; IL-10, interleukin-10; LDL-C, low-density lipoprotein cholesterol; log, logarithmically transformed variable; OR, odds ratio; PASI, Psoriasis Area and Severity Index; Ref., reference category; TNF-α, tumor necrosis factor alpha.

Table S4. Multicollinearity assessment results

Variable	VIF
Age	1.11
Fasting glucose	1.50
HbA1c	1.45
HDL-C	1.09
LDL-C	1.25
Triglycerides, log-transformed	1.19
